# Need for a competency-based approach for the Public Health Workforce training: the ISS proposal: Guglielmo Arzilli

**DOI:** 10.1093/eurpub/ckac131.028

**Published:** 2022-10-25

**Authors:** A Mazaccara, G Privitera, A Di Pucchio, D Barbina, L Bertinato, A Mistretta, M Simonelli, S Caminada, G Arzilli, S Brusaferro

**Affiliations:** 1 Training Office, National Institute of Health, Rome, Italy; 2 Scientific Secretariat to the President Head, National Institute of Health, Rome, Italy; 3 Scientific Communication Unit, National Institute of Health, Rome, Italy; 4 National Center for Global Health, National Institute of Health, Rome, Italy; 5 Department of Public Health and Infectious Diseases, Sapienza University of Rome, Rome, Italy; 6 Department of Translational Research and New Technologies in Medicine and Surgery, University of Pisa, Pisa, Italy; 7 National Institute of Health, Rome, Italy

## Abstract

**Issue:**

The COVID-19 pandemic acted as a catalyst for a reconsidering health care professional workforce (HCPW) competency needed to face future public health challenges, in line with the recent WHO “Global Competency and Outcomes Framework for UHC” document. In this context, in the Italian G20 Presidency framework, the Italian National Institute of Health (ISS), launched a program named “Laboratorium” - recognised in the G20 Declarations of Ministers of Health and Leaders - aimed to the development of training tools suitable for distance learning in whatever context they are placed.

**Description of the problem:**

Common competencies and needs of the HCPW have to be fulfilled through cross-sectional and multilevel training, to overcome the current emergency and be able to face future challenges. The aim is to provide a modular educational approach that is finalized to actively involve the participant: health professionals step in their training pathway according to their knowledge gaps, identify their specific learning objectives and, through a problem-based learning approach, acquire the proper skills for their function and tasks.

**Results:**

We propose a two-tiered approach to training: the development of competence based courses oriented to active learning. A pilot course on Epidemic Intelligence design according to this model is ready to be released. At the same time, a repository of everything available on the web systematically selected from authorised sources by a scientific committee was created to support the training objective. It is characterised by a detailed multilevel tagging that allows an effective modularisation to reach only the content useful for its user.

**Lessons:**

Accessibility to health-related training materials is a required necessity at every level of HCPW. Profiles within healthcare are increasingly heterogeneous and there is a need to focus on disseminating tools that can provide knowledge to address different healthcare scenarios and common purposes.

**Key messages:**

• Future public health challenges underline a need for a competencies-based learning approach in Healthcare Workforce training.

• Italian National Institute of Health propose two methods within the ‘Laboratorium’ project to enable this type of learning: specifically designed courses and a structured document repository.

